# Supplementary material for: Directed Functional Brain Connectivity is Altered in Sub-threshold Amyloid-β Accumulation in Cognitively Normal Individuals
Source: Neurosci Insights. 2023 Mar 29;18:26331055231161625. doi: 10.1177/26331055231161625 (PMC10064157; doi:10.1177/26331055231161625)
Supplement: sj-docx-1-exn-10.1177_26331055231161625 – Supplemental material for Directed Functional Brain Connectivity is Altered in Sub-threshold Amyloid-β Accumulation in Cognitively Normal Individuals [file sj-docx-1-exn-10.1177_26331055231161625.docx]

**Directed functional brain connectivity is altered in sub-threshold amyloid-β accumulation in cognitively normal individuals**

**Supplementary Information**

**Mite Mijalkov^1,*^, Dániel Veréb^1^, Anna Canal-Garcia^1^, Giovanni Volpe^2^, Joana B. Pereira^1,3,*^, Alzheimer’s Disease Neuroimaging Initiative**

^1^ Neuro Division, Department of Clinical Neuroscience, Karolinska Institutet, Stockholm, Sweden.

^2^ Department of Physics, Goteborg University, Gotebörg, Sweden.

^3^ Memory Research Unit, Department of Clinical Sciences Malmö, Lund University, Lund, Sweden.

* Corresponding authors: Mite Mijalkov and Joana B. Pereira,

Address: KI, Dept. NVS, division of clinical geriatrics, Neo 7th floor, Blickagången 16, 141 83 Huddinge, Sweden.

\emails: mite.mijalkov@ki.se // joana.pereira@ki.se


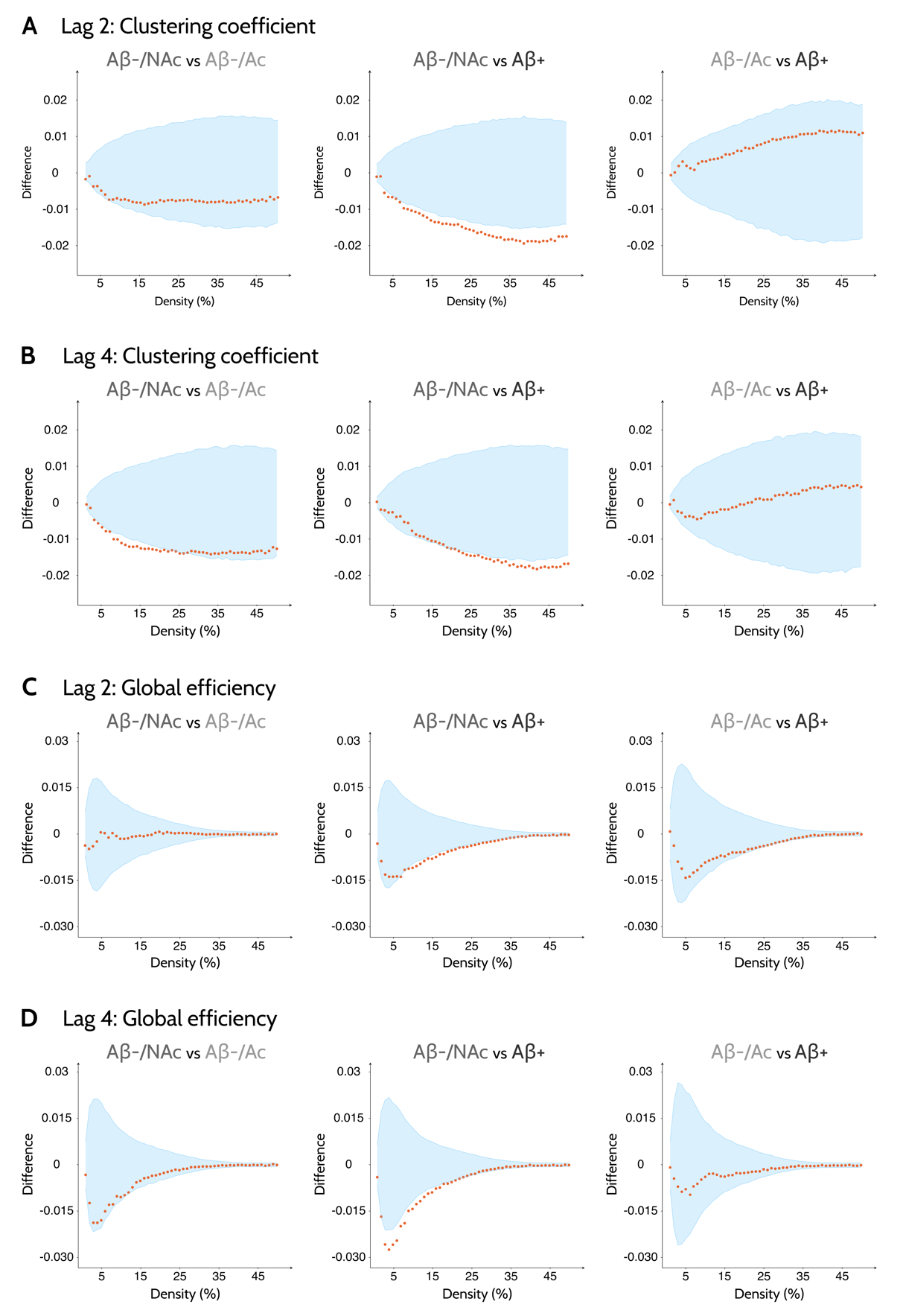


**Fig S1. Differences between different groups in global directed functional network topology.** Between-group differences (calculated as group 2 - group 1 in the figure) in clustering coefficient at A) lag 2 and B) lag 4 as well as in global efficiency at C) lag 2 and D) lag 4. Orange circles represent the differences in the corresponding network measures as a function of network density; the upper and lower bounds of the 95% confidence intervals (CI) are plotted in blue. Differences are considered statistically significant if they fall outside the CIs.
